# Multifocal synchronous renal cell carcinoma of three different histologic subtypes: unusual findings and literature review

**DOI:** 10.1093/jscr/rjac452

**Published:** 2022-09-30

**Authors:** Martha Chavez, Ashka Dharia, Armand Asarian, Philip Xiao

**Affiliations:** Department of Medicine, St George’s University School of Medicine, True Blue, Grenada, WI, USA; Department of Surgery, The Brooklyn Hospital Center, Icahn School of Medicine at Mount Sinai, Brooklyn, NY, USA; Department of Surgery, The Brooklyn Hospital Center, Icahn School of Medicine at Mount Sinai, Brooklyn, NY, USA; Department of Pathology, The Brooklyn Hospital Center, Icahn School of Medicine at Mount Sinai, Brooklyn, NY, USA

## Abstract

We present a case of three multifocal synchronous ipsilateral clear cell, papillary type 1 and papillary type 2 renal cell carcinoma (RCC), with papillary type 2 RCC appearing bilaterally. With review of the literature, it was determined that multifocal synchronous RCC subtypes and bilateral renal tumors are both rare in occurrence.

## INTRODUCTION

Renal cell carcinoma (RCC) is the most common tumor of the kidney, and includes clear cell and papillary subtypes among others [1]. Histologically, clear cell RCC presents with cells with lipid and glycogen rich cytoplasm [2]. Papillary RCC, however, is further classified into type 1 and type 2 [2]. Type 1 presents with basophilic cells with scarce clear cytoplasms and hyperchromatic nuclei surrounding the basal membrane [2]. Type 2 presents with papillae covered by cells with abundant granular eosinophilic cytoplasm with prominent nucleoli associated with areas of necrosis [2].

The presence of histologically different subtypes of renal tumors in the same kidney is rare, with most case reports describing up to two subtypes [3]. Furthermore, bilateral renal tumors are also rare [4]. We report a rare case of three multifocal synchronous ipsilateral RCC subtypes, which included clear cell, papillary type 1 and papillary type 2, with papillary type 2 presenting bilaterally.

## CASE PRESENTATION

A 53-year-old male who underwent an abdominal computed tomography scan with intravenous contrast during evaluation for flank pain was revealed to have multiple tumors in the right kidney and one tumor in the left kidney. A robot-assisted laparoscopic partial nephrectomy of the left kidney and radical nephrectomy of the right kidney were performed.

Upon gross examination, the right kidney had multiple separate gray-yellow tumors that measured up to 4.2 x 3.6 x 3.5 cm. One tumor in the upper pole was 1.8 cm and one tumor in the posterior mid pole was 1.2 cm. The largest tumor was bulging towards the anterior surface of the kidney and 1.6 cm away from the hilar margin. The left kidney had one tumor that measured 8.0 x 6.0 x 5.0 cm and there was a capsular disrupted area that measured 4.0 x 1.6 cm.

**Figure 1:**
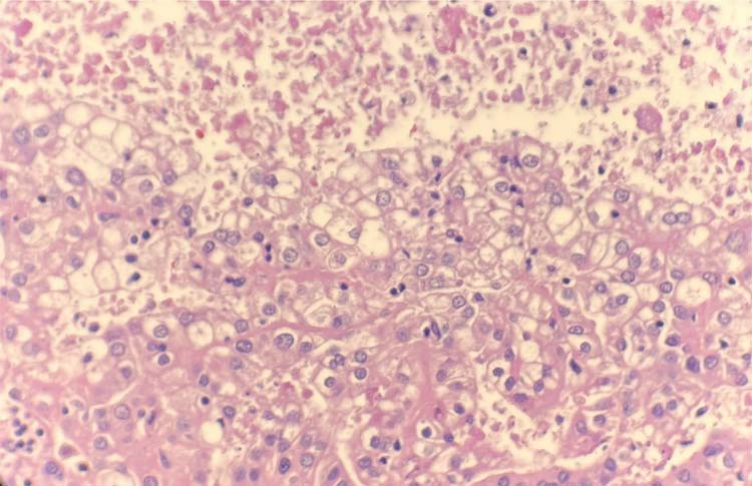
Microscopic examination reveals compact tumor nests and sheets of cells with clear cytoplasm and distinct membrane. HE stain 40x.

**Figure 2:**
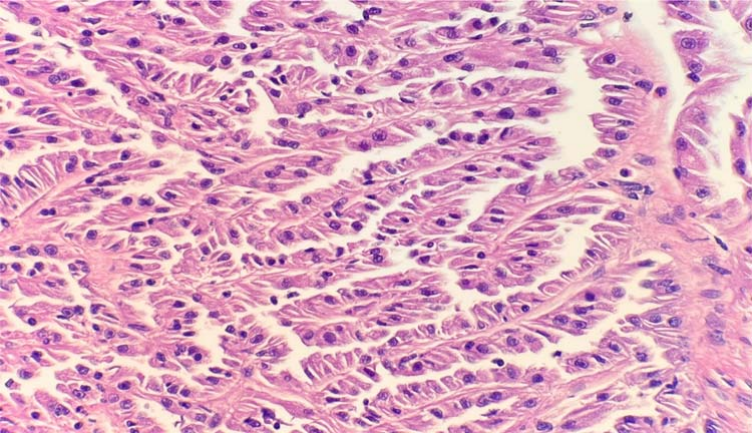
Microscopic examination reveals small cuboidal cells arranged in a single layer on papillary cores. HE stain 40x.

**Figure 3:**
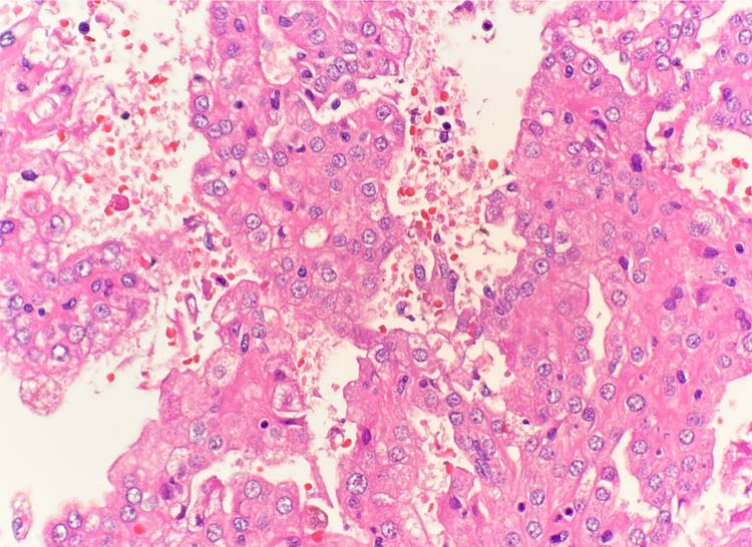
Microscopic examination reveals pseudostratified tumor cells with papillary architecture, abundant eosinophilic, atypical nuclei and prominent nucleoli. HE stain 40x.

**Figure 4:**
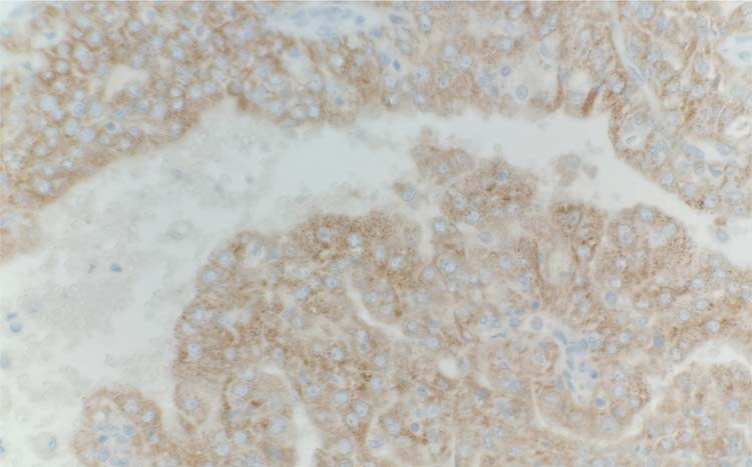
Immunohistochemical stain reveals that tumor cells are positive for alpha-methyacyl-CoA racemase. IHC stain 40x.

Microscopic examination revealed that clear cell RCC (Fig. 1), papillary RCC type 1 (Fig. 2), and papillary RCC type 2 (Fig. 3) were present in the right kidney, and papillary RCC type 2 was present in both kidneys. Immunostaining showed that tumor cells were positive for alpha-methyacyl-CoA racemase (Fig. 4), CD10, CK7, and vimentin, and negative for CD117.

## DISCUSSION

Renal tumors of varying histology in the same kidney and renal tumors presenting bilaterally are rare [3, 4]. To our knowledge after reviewing the literature, this is the first report of multifocal synchronous ipsilateral RCC of three different histologic subtypes, which included clear cell, papillary type 1 and papillary type 2, with papillary type 2 presenting bilaterally. There have been only a few case reports of two synchronous RCC subtypes appearing in the same kidney, and currently one case report of multifocal RCC with synchronous tumors of three different histologic subtypes in the same kidney.

Etiology for RCC includes smoking, obesity and hypertension [5]. The most common symptoms in patients with RCC are hematuria, flank pain and mass effect [6]. RCC can be treated with partial or radical nephrectomy, ablation or active surveillance [1]. Histopathological confirmation of malignancy is obtained with renal core biopsy or on the nephrectomy specimen [1]. Histological classification of RCC is important due to therapeutic and prognostic implications depending on the histological subtype. Preoperative radiological characterization followed by confirmatory percutaneous biopsy is useful in cases of poor surgical condition, metastatic disease and determining the type of cancer [4].

There is currently insufficient data for different subtypes of RCC in the same kidney and bilateral renal tumors in terms of survival or oncologic survey. In fact, the origin and evolution of these two rare entities remain elusive. Many patients with multiple renal tumors have an underlying hereditary renal tumor syndrome (HRTS), especially if the patient has bilateral renal tumors [7]. However, most bilateral RCCs are sporadic and do not reveal a hereditary pattern [8]. Clinical implication and information about long term outcome for patients with bilateral renal tumors and patients with multiple tumors in the same kidney is limited, so studies are needed to compare disease courses and determine a management approach.

Surgical methods for patients with ipsilateral or bilateral renal tumors include radical nephrectomy in cases of large or multiple tumors, partial nephrectomy for the contralateral kidney with low tumor burden, or bilateral partial nephrectomy for bilateral small tumors [4]. This patient was treated with radical and partial nephrectomy. It is possible that this patient has a worse prognosis or higher likelihood of recurrence given the presentation of three synchronous subtypes of RCC, with one present bilaterally. It is also possible that this patient has a HRTS given the presence of multiple renal tumors.

Patients with HRTS are at an increased risk for the development of additional renal tumors, even when the original tumor has been resected [7]. These patients typically require specialized nephron-sparing surgery and careful surveillance [7]. Additionally, if the patient needs adjuvant treatment, it may be difficult to determine the regimen due to the varying nature and location of each subtype. Careful monitoring and close follow up of patients with different subtypes of RCC in the same kidney and patients with bilateral renal tumors are important because information is not widely available.

## CONCLUSION

More than one subtype of RCC in the same kidney is rare, with most cases presenting with up to two subtypes. Furthermore, bilateral renal tumors are also rare. In this rare case, three multifocal synchronous ipsilateral RCC subtypes were present which included clear cell, papillary type 1 and papillary type 2, with papillary type 2 presenting bilaterally. There is currently insufficient data to compare different RCC subtypes in the same kidney and bilateral tumors in terms of survival or oncologic survey due to these being rare occurrences. The possibility for HRTS in these patients should be considered, and further research is required in order to have better diagnostic and treatment guidelines.

## CONFLICT OF INTEREST STATEMENT

None declared.

## FUNDING

None.
